# Understanding Intra- and Inter-Species Variability in Neural Stem Cells’ Biology Is Key to Their Successful Cryopreservation, Culture, and Propagation

**DOI:** 10.3390/cells12030488

**Published:** 2023-02-02

**Authors:** Klaudia Radoszkiewicz, Katarzyna Jezierska-Woźniak, Tomasz Waśniewski, Anna Sarnowska

**Affiliations:** 1 Translational Platform for Regenerative Medicine, Mossakowski Medical Research Institute, Polish Academy of Sciences, 02-106 Warsaw, Poland; 2 Department of Neurosurgery, Laboratory for Regenerative Medicine, Stem Cells Bank, University of Warmia and Mazury in Olsztyn, 10-720 Olsztyn, Poland; 3 Department of Obstetrics and Gynaecology, School of Medicine, Collegium Medicum, University of Warmia and Mazury, 10-561 Olsztyn, Poland

**Keywords:** neural stem cells, intra-species variability, inter-species variability, cryopreservation, long-term culture, human, rodents, medium composition, growth factors

## Abstract

Although clinical trials on human neural stem cells (hNSCs) have already been implemented in the treatment of neurological diseases and they have demonstrated their therapeutic effects, many questions remain in the field of preclinical research regarding the biology of these cells, their therapeutic properties, and their neurorestorative potential. Unfortunately, scientific reports are inconsistent and much of the NSCs research has been conducted on rodents rather than human cells for ethical reasons or due to insufficient cell material. Therefore, a question arises as to whether or which conclusions drawn on the isolation, cell survival, proliferation, or cell fate observed *in vitro* in rodent NSCs can be introduced into clinical applications. This paper presents the effects of different spatial, nutritional, and dissociation conditions on NSCs’ functional properties, which are highly species-dependent. Our study confirmed that the discrepancies in the available literature on NSCs survival, proliferation, and fate did not only depend on intra-species factors and applied environmental conditions, but they were also affected by significant inter-species variability. Human and rodent NSCs share one feature, i.e., the necessity to be cultured immediately after isolation, which significantly maintains their survival. Additionally, in the absence of experiments on human cells, rat NSCs biology (neurosphere formation potential and neural differentiation stage) seems closer to that of humans rather than mice in response to environmental factors.

## 1. Introduction

**Figure 1:**
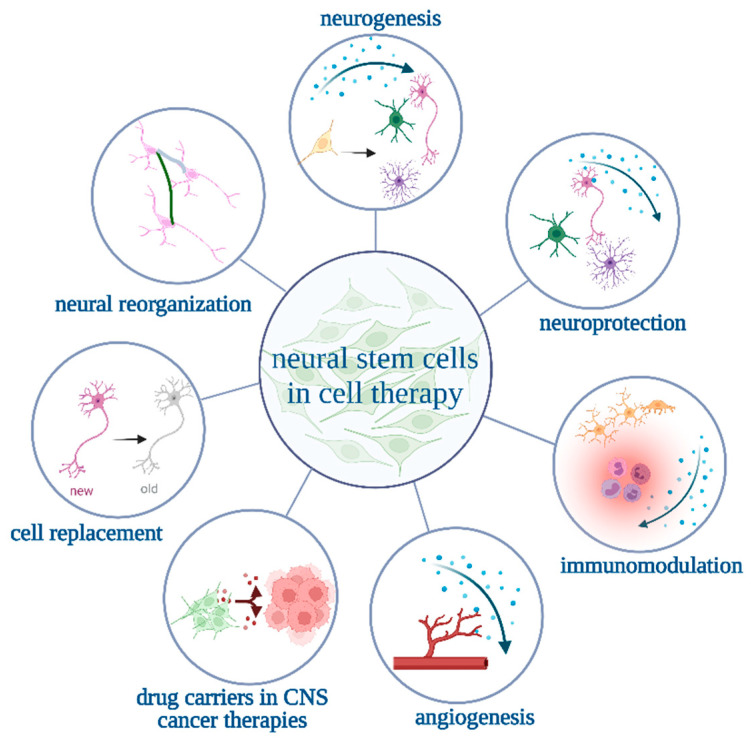
Possible benefits of neural stem cells’ (NSCs) use in clinical trials suggest the need to conduct further NSC studies at the preclinical level.

Over the last four decades of studies on neural stem cells’ (NSCs) clinical applicability, researchers from different laboratories have optimized culture conditions which facilitate long-term culture and further cell differentiation. A considerable interest in NSCs is mainly aroused by promising results obtained in patients with neuropsychiatric disorders and a variety of neurological diseases, such as amyotrophic lateral sclerosis, multiple sclerosis, glioma, Parkinson’s disease, and ischemic stroke, which in most cases still need successful treatment [1]. This is caused by a variety of beneficial NSC properties (Figure 1). In comparison to other cell types, NSCs demonstrate a restricted ability to differentiate into mature, functional cells which can replace the injured ones, and they show a minimal risk of tumorigenesis [2,3,4]. What is more, they can migrate into the damaged areas and promote tissue repair by secreting pro-neurogenic and pro-angiogenic factors [5,6,7,8,9,10,11,12,13]. Human neural stem cells’ (hNSCs) potential has not only been detected in trophic support but also in a cell replacement strategy (neurorestoration) after damage [12]. Moreover, hNSCs derived from the human olfactory bulb (hOBNSCs) seem to represent a new approach for anticancer therapy within the central nervous system, as their potential to be used as a carrier/vehicle of anticancer agents has been indicated [14]. In addition, it has been observed that the use of some factors could enhance their therapeutic properties. For example, recent suggestive results of the Alzheimer’s disease rat model study indicated that transplanted NSCs’ further differentiation and integration with the host tissue could be enhanced by the previous administration of rosemary oil [15].

Despite the impressive variety of the aforementioned benefits, the attempts to carry out cell-based therapy with NSCs have revealed several limitations [12]. Even at the preclinical level, the interpretation of the results obtained by different laboratories could be hampered because of several variables, such as different medium composition, 2D (in monolayer) or 3D (as neurospheres) spatial culture conditions, dissociation methods, and/or the species used for analyses. Moreover, it is difficult to assess which NSC origin type would be the most efficient for cell therapy [16,17]. The question also arises as to which conclusions regarding the isolation, cell survival, proliferation, or cell fate observed *in vitro* in rodent NSCs are appropriate and sufficient to be introduced into clinical applications.

In our study, we compared intra-species and inter-species variability in NSCs’ biology as a response to different cryopreservative, spatial, nutritional, and dissociation conditions. For the analysis, fetal hNSCs obtained from the University of Warmia and Mazury in Olsztyn were used [18]. Rodent NSCs were derived from the most commonly used Wistar rats and C57BL/6J-type mice.

**Figure 2:**
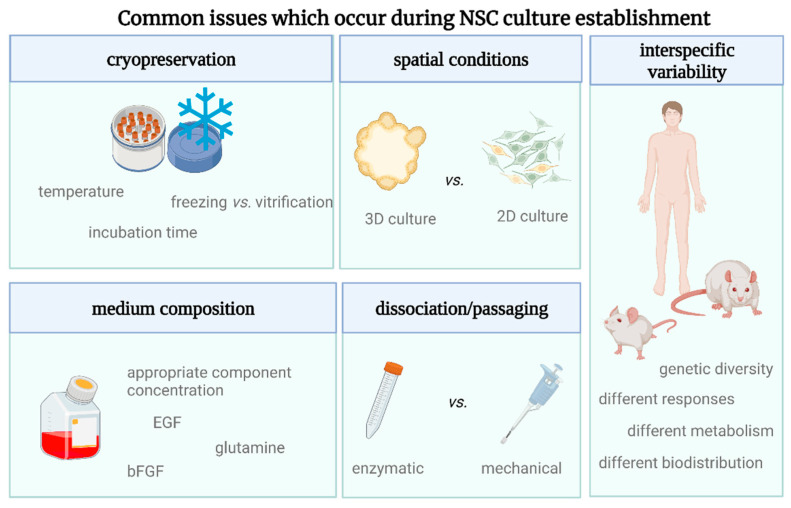
A variety of culture establishment options regarding NSCs’ cultivation.

Throughout our research, we focused on the differences in the cell fate, which might depend even on negligible environmental variables, and we underlined the problems with the translation of the results from rodent into human cells (Figure 2).

## 2. Materials and Methods

### 2.1. NSC Isolation

#### 2.1.1. hNSCs’ Isolation

hNSCs were obtained from the Stem Cell Research Laboratory, Department of Neurosurgery, University of Warmia and Mazury in Olsztyn, Poland, where the material was collected and prepared according to the protocol developed by Prof. Vescovi’s group [19,20,21], slightly modified for dissociation by means of Accutase Cell Detachment Solution (Beckton Dickinson, Franklin Lakes, NJ, USA). The neural tissue was obtained from the whole brain in 2020, from fetuses after spontaneous miscarriages from 10 and 18 weeks of gestation (mixed XX and XY). The study was approved by the Bioethical Committee of the School of Medicine, University of Warmia and Mazury in Olsztyn, Poland (ethical approval No. 15/2018 and No. 19/2018, both in May 2018). One part of the cells was cultured before cryopreservation and the other part was cryopreserved directly after the isolation. After isolation, to identify the undifferentiated state of NSCs, immunocytochemical staining of anti-Nestin and anti-SOX-2 was performed. To study the multipotentiality of NSCs, their differentiation potential into glial or neuronal progenies was analyzed after growth factor removal. The cells were plated into 24-well plates in the medium supplemented without epidermal growth factor (EGF) and basic fibroblastic growth factor (bFGF) for 7 days. The presence of the astrocytic marker GFAP, neuronal marker β-tubulin III, and oligodendrocytic marker O4 were analyzed by immunofluorescence (IF).

#### 2.1.2. Mouse Neural Stem Cells (mNSCs) and Rat Neural Stem Cells (rNSCs) Isolation

For this experiment, newborn Wistar rats and C57BL/6J-type mice from the Mossakowski Medicine Research Institute Animal Breeding House were used.

**Figure 3:**
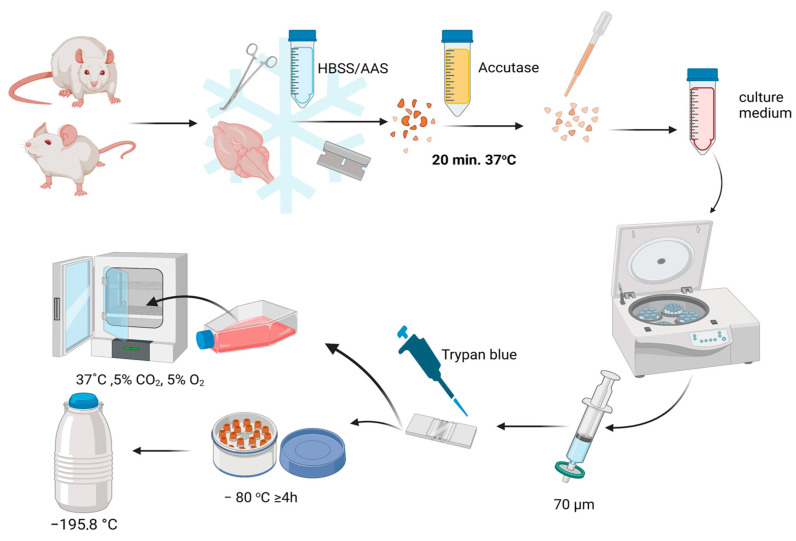
rNSCs and mNSCs isolation procedure.

All macro- and micro-dissection procedures were performed on ice in a careful and swift manner (Figure 3). After decapitation, murine/rat brains were extracted, and the area around the subventricular zone (SVZ) was isolated. To remove the pia mater and blood vessels, an additional set of microdissection instruments was used under a binocular. The samples were mechanically defragmented using a razor blade, collected in a 15 mL centrifuge tube, and centrifugated (5 min, 200× *g*). Then, the pellet was digested in Accutase^®^ solution (Beckton Dickinson) for 20 min, at 37 °C. After every 5 min of digestion, the sample was gently triturated using a Pasteur Pipette. Next, the samples were centrifugated again and the pellets were resuspended in a freshly prepared culture medium, filtered in a 70 µm cell filter, and 10 µL of suspension aliquots were mixed with 10 µL of Trypan blue (Sigma-Aldrich; Merck; Irvine; United Kingdom) in a continuous manner at room temperature (RT) to calculate cell viability. The obtained cells were seeded in non-adhesive T75 flasks (Nunc, Thermo Fischer; Rochester, NY, USA) at a density of 2 × 10^5^ cells/mL and incubated at 37 °C with 5% CO_2_ and 5% O_2_ or cryopreserved in 1.5 mL cryovials, at high density in a medium consisting of 50% basal medium (cells+culture medium), 20% fresh culture medium, 20% platelet lysate (Macopharma, Tourcoing, France), and 10% DMSO (Sigma-Aldrich, St Louis, MO, USA). The vials placed in a freezing container (Mr. Frosty, Thermo Fisher Scientific, Rochester, NY, USA) and were transferred to a −80 °C freezer for at least 4 h in our laboratory, where they were stored in liquid nitrogen. The same identification of the undifferentiated state and multipotentiality of rodent NSCs as was shown for hNSCs was performed.

### 2.2. Cell Culture

#### 2.2.1. Thawing

The thawing procedure included immediate warming of cryotubes (maximum 2 min at 37 °C, in a water bath), the cells’ suspension in a standard culture medium, and centrifugation (to eliminate the toxic impact of DMSO (Sigma-Aldrich)). Then, the cells from the pellet were suspended in a fresh standard culture medium. Next, the number of cells was counted using the Bruker chamber.

#### 2.2.2. Basic Culture

The optimal medium composition and seeding density for NSCs was determined on the basis of the latest relevant literature (Table 1).

Next, the obtained cultures were placed in humidified incubators under 5% O_2_ and 5% CO_2_ conditions at 37 °C. The medium was replaced every 3 days. When the cell culture was subconfluent (in 2D culture) or the neurospheres (3D) gained the desired diameter, the cells were detached (2D) or dissociated (3D) with Accutase^®^ (Accutase Cell Detachment Solution, Beckton Dickinson, Franklin Lakes, NJ, USA). In case of further proliferation, lactate dehydrogenase (LDH) and senescence were used for NSCs’ differentiation state analysis (Results Section 3.2, Section 3.3, Section 3.4 and Section 3.5), and the cultures were sub-cultured, from four to six passages, before starting the aforementioned experiments, in order to enrich the culture in NSCs.

### 2.3. Live/Dead Assay

A live–dead assay (Invitrogen™) was used according to the manufacturer’s instructions. Briefly, the medium was removed, and the cells were washed with phosphate buffer saline (PBS, Sigma-Aldrich) followed by incubation with the dyes. Live cells (stained with calcein-AM) and dead cells (stained with ethidium homodimer-1 (EthD-1)) were visualized using a Zeiss Axiovert A.1 fluorescent microscope (Carl Zeiss, Oberkochen, Germany). The percentage of living and dead cells was calculated by determining the percentage of calcein-AM/EthD-1-positive cells in the total number of cells.

### 2.4. Proliferation Assay

To estimate the cell proliferation rate, PrestoBlue™ Cell Viability Reagent (Invitrogen, Thermo Fischer, Rochester, NY, USA) was used according to the manufacturer’s protocol. The cells were incubated with this reagent for 2 h in the dark, on Day 1, Day 5, and Day 7 of 2D culture performed on a 96-well poly-L-lysine/laminin-coated plate. The cells were seeded at a density of 1 × 10^4^ cells/well. The starting point (Day 1 measurement) was assumed to equal 100%. Absorbance was measured using a microplate reader, Omega Plate Reader (BMG LABTECH), at a 590–600 nm wavelength.

### 2.5. Lactate Dehydrogenase Assay

Cell viability was measured using the LDH assay and trypan blue (Sigma-Aldrich) staining was used at the cell count stage (Takara). A colorimetric assay kit (Takara Bio Inc., Kusatsu, Japan) was used to quantify LDH release from cultured NSCs, in line with the producer’s instructions. Absorbance was measured using a microplate reader, Omega Plate Reader (BMG LABTECH), at a 490–690 nm wavelength. The final LDH release was calculated according to the following formula:

LDH release (%) = 100 × (experimental LDH release-culture medium background)/(maximum LDH release-culture medium background).

### 2.6. Senescence Assay

The senescence analysis was performed with a CellEvent™ Senescence Green Detection Kit (Thermo Fischer Scientific, Waltham, MA, USA) according to the manufacturer’s protocol. In brief, the cells were seeded on 96-well plates (Nunc, Thermo Fischer Scientific), washed with PBS (Sigma-Aldrich), fixed with a fixation solution (2% PFA (Sigma-Aldrich) in PBS (Sigma-Aldrich)), and incubated in a working solution without CO_2_ at 37 °C for 2 h. Then, the cells were washed with PBS. The fluorescence intensity was measured using a microplate reader, Omega Plate Reader (BMG LABTECH).

### 2.7. Immunocytochemistry 2D

For immunocytochemical analysis, the cells cultured in 2D and 3D conditions were seeded on 24-well plates (Nunc, Thermo Fischer Scientific). The seeding number of cells was 7 × 10^4^ per each 13 mm-diameter poly-L-lysine/laminin-coated coverslip. The 2D culture cells were gently washed in PBS (Sigma-Aldrich), fixed with 4% PFA (Sigma-Aldrich) in PBS (Sigma-Aldrich) for 15 min at RT, and again washed in PBS (Sigma-Aldrich). To detect intracellular proteins and to permeabilize cell membranes, 0.2% Triton X-100 (Sigma-Aldrich) was used. Subsequently, a mixture of 10% goat serum (Gibco) and 1% bovine serum albumin (Sigma-Aldrich) was applied for one hour to block nonspecific binding. Next, the samples were washed in PBS (Sigma-Aldrich) and incubated with primary antibodies overnight at 4 °C. For each variant, a negative control was performed. The cells were washed in PBS (Sigma-Aldrich), and the incubation with secondary antibodies was performed in the dark for 2 h. After the cells were washed again in PBS (Sigma- Aldrich), the nuclei were stained using Hoechst 33342 (Sigma-Aldrich) for 15 min. All the samples were analyzed with the LSM 780 confocal laser scanning system and ZEN software (Carl Zeiss). Quantitative analysis was performed as a relation of positive cells to a total cell number (50 cells per repetition). Each variant had 3 repetitions.

### 2.8. Developing a New Procedure of Immunocytochemical Staining for Better hNSCs Neurosphere (3D) Immunofluorescent Visualization

The next step was to develop a protocol for neurosphere staining (3D conditions) which would allow for the cell staining and visualization in the core of the neurosphere, thus enabling the analysis to be performed without the need for cryosection. The neurospheres were fixed in 4% PFA (Sigma-Aldrich) in PBS (Sigma-Aldrich) for 15 min at RT, then washed with 0.1% Triton X-100 (Sigma-Aldrich) in PBS (Sigma-Aldrich). The next step involved blocking non-specific bonds for at least 12 h using a blocking mixture (made with 6% BSA solution (Sigma-Aldrich), 0.1% Triton X-100 (Sigma-Aldrich) in PBS (Sigma-Aldrich) at RT. The neurospheres were washed with 0.1% Triton X-100 (Sigma-Aldrich) in PBS (Sigma-Aldrich) and 24 h incubation with primary antibodies (Table 2) was performed at 2–8 °C. After the 0.1% Triton X-100 (Sigma-Aldrich) wash in PBS (Sigma-Aldrich), 24 h incubation with secondary antibodies (Table 3) at RT was carried out in the dark. Next, the neurospheres were washed again with 0.1% Triton X-100 (Sigma-Aldrich) in PBS (Sigma-Aldrich) and stained with 1% Hoechst 33342 (Sigma-Aldrich) solution for 30 min to visualize the nuclei. After washing the neurospheres with 0.1% Triton X-100 (Sigma-Aldrich) in PBS (Sigma-Aldrich), 20 µL of the Triton/PBS solution with the stained neurospheres was transferred to the surface of the slide, drawing off the solution with due care so as not to pull the spheres away. A single drop of the mounting medium was applied and closing with a 14 mm-diameter coverslip was performed. The slides were left at RT for overnight drying.

### 2.9. Statistical Analysis

Raw data statistical analysis was performed with GraphPad Prism 7 software. The results are presented as mean and standard deviation. To conduct multi-group comparisons, a one-way analysis of variance (ANOVA) was used, followed by the Tukey test as a post hoc statistical analysis for each group. The values were considered significant at *p* < 0.05.

## 3. Results

### 3.1. The Effect of Direct Cryopreservation on Human and Rodent NSCs’ Viability and Growth Potential

#### 3.1.1. Intra-Species Variability

**Figure 4:**
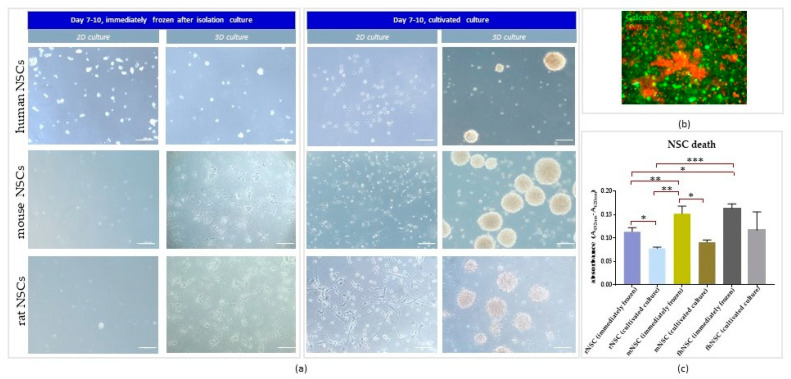
The effect of freshly isolated NSCs’ cryopreservation on their viability and growth potential. (**a**) NSCs on days 7–10 of 2D and 3D culture after the isolation: NSCs frozen right after isolation (on the left) and NSCs after the standard cultivation procedure (on the right). (**b**) Immunofluorescent analysis of dead (stained with red Edth-1) and live (stained with green calcein) immediately frozen hNSCs. (**c**) Immediately frozen and cultivated NSCs’ death depending on the species after 24 h of 3D culture. Cell culture conditions: 5% O_2_, 5% CO_2_, 37 °C. N = 3. The results of all experiments presented above are expressed as mean values of three experiments ± SEM. The differences were considered statistically significant when the *p*-value < 0.05. Statistical significance level: * for 0.01 < *p* < 0.05, ** for 0.001 < *p* < 0.01, and *** for 0.0001 < *p* < 0.001.

hNSCs which were cryopreserved directly after the isolation showed a reduced viability (higher LDH release in the first 24 h of culture) and growth potential after thawing than the cultivated ones (Figure 4c), regardless of the type of culture (2D or 3D). These cells needed a much longer timespan to form spheres or adhere to plastic than the cells cultivated directly after isolation. The defrosted hNSCs began to form neurospheres after 10–15 days, eventually reaching a diameter of 100–120 µm, as opposed to cultivated hNSC which needed 7–10 days to demonstrate the same effect (Figure 4a).

Despite the optimization of the thawing protocol, such as: omitted centrifugation of cells before their resuspension in the basic medium (to avoid possible mechanical damage to the cells), the first change of the medium performed after the second day of culture (to reduce stress after thawing), and a higher concentration of glutamine in the medium (to possibly increase their proliferative potential), it was not possible to obtain the sufficient number of cells to establish long-term cultivation and expansion (Figure 4b).

NSCs isolated from both mice and rats and immediately cryopreserved showed a significantly reduced viability after thawing, regardless of the type of culture (2D or 3D), which is consistent with the observations made on hNSC culture (Figure 4c) that needed approximately 7–10 days longer to reach the stage of development observed on Day 7 in freshly-isolated culture (Figure 4a).

#### 3.1.2. Inter-Species Variability

No differences between rNSCs and mNSCs in cell fate were observed with regard to the post-isolation protocol. Despite no statistically significant difference, the tendency observed in human NSCs seemed to be similar in rodent NSCs (Figure 4).

### 3.2. The Influence of the Medium Composition (Growth Factors, Glutamine) on the Viability, Proliferation, and Senescence of Human and Rodent NSCs

The viability, proliferation, and cell senescence were analyzed after 1, 3, and 7 days of cell culture using the following medium variants:

- **Full medium**—the medium with 20 ng/mL of basic fibroblast growth factor (bFGF) and epidermal growth factor (EGF), which is a control for our experiments.
- **FGF**—standard medium, with bFGF as the only growth factor (without EGF).
- **EGF**—standard medium, with EGF as the only growth factor (without bFGF).
- **FGF20/EGF10**—standard medium, with 20 ng/mL of bFGF and 10 ng/mL of EGF.
**-GFs**—standard medium without bFGF and EGF.
**-GFs, Gln**—standard medium without bFGF, EGF, and glutamine.
**-Gln**—standard medium without glutamine.

#### 3.2.1. Intra-Species Variability in 2D

**Figure 5:**
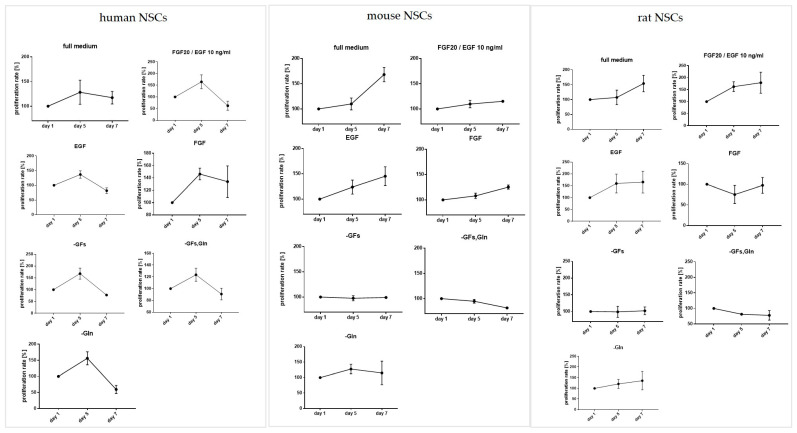
The influence of culture medium composition on the cell proliferation rate on Days 1, 5, and 7 in relation to the cells’ origin. Cell culture conditions: 5% O_2_, 5% CO_2_, 37 °C. N = 3. The results are presented as mean values of three experiments ± SEM.

Only in the full medium was hNSC proliferation potential maintained at a constant level (117 ± 12%) (Figure 5). In each incomplete variant of the medium, the proliferation significantly decreased, especially in the cells cultured in the media without GFs, GFs and Gln (90 ± 10 %), and without glutamine (up to 59 ± 12%).

**Figure 6:**
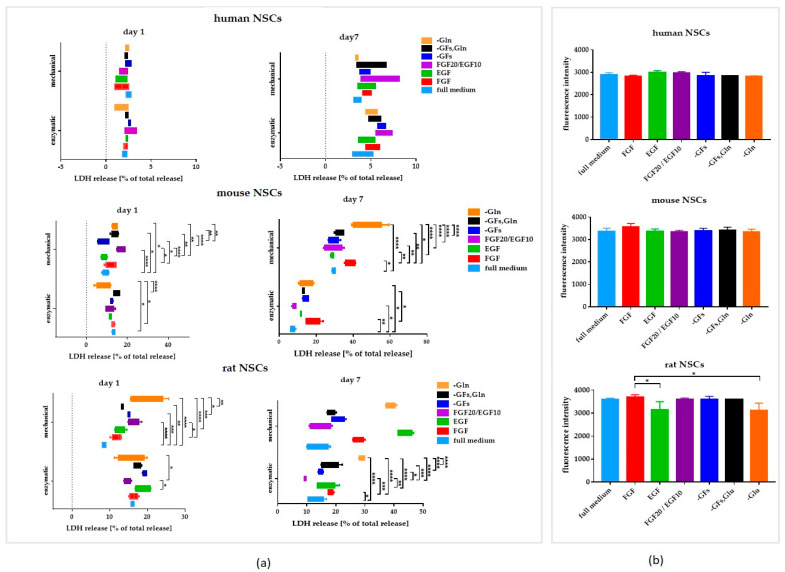
The influence of the medium composition on the viability and senescence of NSCs. (**a**) LDH release of NSCs on Day 1 and Day 7 of culture after enzymatic/mechanical dissociation. (**b**) Senescence activity of NSCs after Day 7 of culture. Cell culture conditions: 5% O_2_, 5% CO_2_, 37 °C, N = 3. The results of all presented experiments are expressed as mean values of three experiments ± SEM. The differences were considered statistically significant when the *p*-value < 0.05. Statistical significance level: * for 0.01 < *p* < 0.05, ** for 0.001 < *p* < 0.01, *** for 0.0001 < *p* < 0.001, and **** for *p* < 0.0001.

The medium composition did not influence hNSCs’ viability in a significant manner (LDH test) (Figure 6a).

Next, the senescence in hNSCs was analyzed (Figure 6b) and no significant changes were observed between the presented culture variants.

mNSC culture was found less demanding and the cells were also able to proliferate in the incomplete medium (Figure 5). After 7 days, a significant decrease in proliferation was noted only for the cells cultured in the medium without growth factors and glutamine (81.5 ± 1%).

On Day 1 of culture, the most substantial changes in cell viability were seen for the -Gln culture medium variant. The cell viability was more elevated (9 ± 3% of total LDH release) than in the remaining variants, especially in comparison to the full medium (13 ± 1% of total LDH release), FGF (13 ± 1% of total LDH release), and the -GFs, Gln (15 ± 2% of total LDH release) variant (Figure 6a). However, the most significant differences in cell viability were seen after 7 days of culture—the lowest viability was observed in the glutamine-free medium (15 ± 4% of total LDH release) and in FGF medium (12 ± 0.1% of total LDH release).

The senescence observed in mNSCs did not significantly differ between the variants (Figure 6b).

After 7 days of culture, rNSCs’ proliferation decreased in the medium without glutamine and without growth factors (78 ± 15%) (Figure 5).

The lowest viability was recorded for rNSCs cultured in the medium without glutamine, both after Day 1 (15 ± 4% of total LDH release) and Day 7 (30 ± 1% of total LDH release) of culture (Figure 6a). For both time points, low cell viability was observed for the FGF20/EGF10 variant (15 ± 8% and 9 ± 0.5% of total LDH release for Days 1 and 7, respectively) (Figure 6a).

Significantly lower cell senescence was observed in the EGF and -Gln media than in the FGF medium (Figure 6b).

#### 3.2.2. Inter-Species Variability in 2D

**Figure 7:**
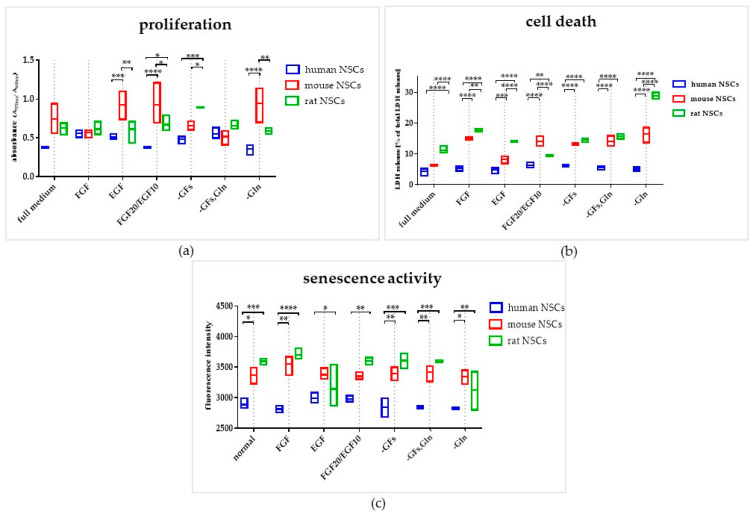
The influence of the medium composition on proliferation, viability, and senescence of 2D NSCs. (**a**) Proliferation of NSCs on Day 7 of culture. (**b**) Cell death of NSCs after Day 7 of culture. (**c**) Senescence activity of NSCs after Day 7 of culture. Cell culture conditions: 5% O_2_, 5% CO_2_, 37 °C, n = 3. The results of all presented experiments are expressed as mean values of three experiments ± SEM. The differences were considered statistically significant when the *p*-value < 0.05. Statistical significance level: * for 0.01 < *p* < 0.05, ** for 0.001 < *p* < 0.01, *** for 0.0001 < *p* < 0.001, and **** for *p* < 0.0001.

The proliferative potential of mNSCs was significantly greater in the media with EGF, FGF20/EGF10, and without glutamine in comparison to hNSCs and rNSCs (Figure 7a). The hNSC proliferation potential was the lowest in comparison to all tested species in most medium variants, except for the FGF medium and -GFs/Gln medium.

However, hNSC demonstrated the best viability regardless of the medium composition, whereas the lowest viability was observed in rNSC culture (Figure 7b). The cell death was positively correlated with the cell senescence for all NSC cultures.

The senescence activity was significantly elevated in all culture variants of rodent NSCs in comparison to hNSCs (Figure 7c).

#### 3.2.3. 3D Cells’ Response to Medium Conditions

**Figure 8:**
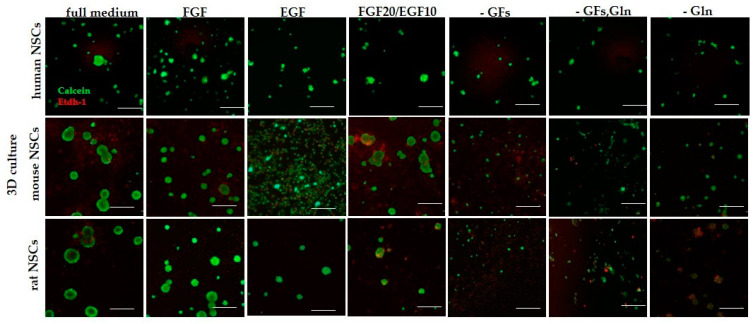
Fluorescence microscopic images of 3D NSCs cultured in different media and spatial conditions, on Day 7 of culture. Cells were stained with calcein (green) and EthD-1 (red) staining. Cell culture conditions: 5% O_2_, 5% CO_2_, 37 °C. Scale: 100 µm.

In 3D culture, hNSCs were observed to create neurospheres in all medium variants. Their growth was slower and the final diameter was smaller in the medium without FGF, without both growth factors, as well as without growth factors and glutamine (Figure 8 and Figure 13). This observation was shown to be repeated even more markedly with regard to both rodent cultures. A strong disability to form neurospheres cultured in a medium without growth factors and glutamine was recorded in both mNSCs and rNSCs. Furthermore, we noticed a reduced number of neurospheres formed by mNSCs treated with the medium without FGF. However, the impact of FGF absence was not as pronounced in rNSCs.

### 3.3. Comparison of Dissociation Methods—Enzymatic vs. Mechanical

**Figure 9:**
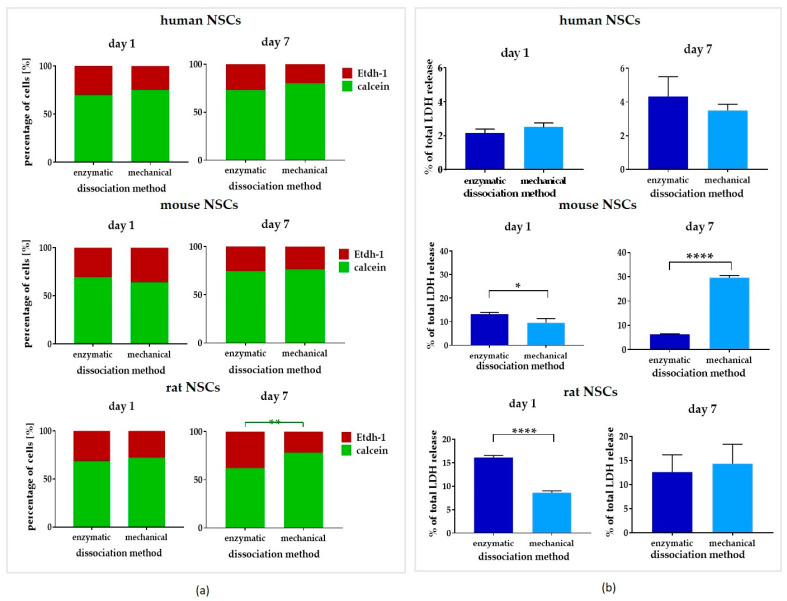
The influence of dissociation methods—enzymatic and mechanical—(**a**) on NSCs’ calcein+ and Etdh-1+ cells’ presence on Day 1 and Day 7 of culture, and (**b**) on NSCs’ LDH release on culture Days 1 and 7. Cell culture conditions: 5% O_2_, 5% CO_2_, 37 °C, N = 3. The results of all presented experiments are expressed as mean values of three experiments ± SEM. The differences were considered statistically significant when the *p*-value < 0.05. Statistical significance level: * for 0.01 < *p* < 0.05, ** for 0.001 < *p* < 0.01, and **** for *p* < 0.0001.

**Figure 10:**
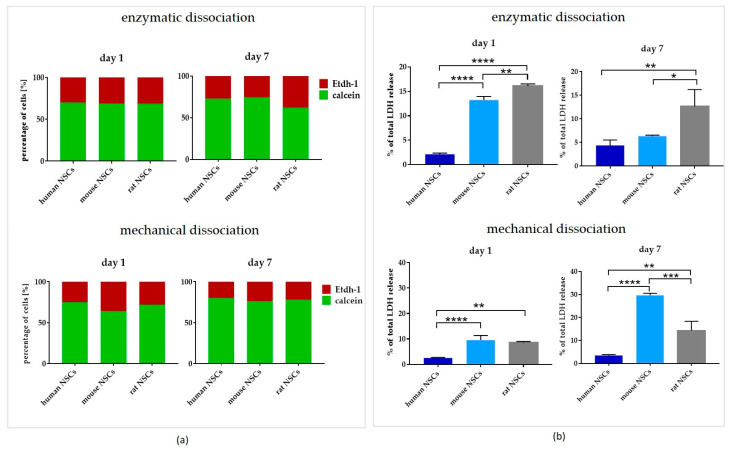
The influence of dissociation methods—enzymatic and mechanical—(**a**) on NSCs’ calcein+ and Etdh-1+ cells’ presence on Day 1 and Day 7 of culture, and (**b**) on NSCs’ LDH release on culture Days 1 and 7. Cell culture conditions: 5% O_2_, 5% CO_2_, 37 °C, n = 3. The results of all presented experiments are expressed as mean values of three experiments ± SEM. The differences were considered statistically significant when the *p*-value < 0.05. Statistical significance level: * for 0.01 < *p* < 0.05, ** for 0.001 < *p* < 0.01, *** for 0.0001 < *p* < 0.001, and **** for *p* < 0.0001.

To expand the 3D culture, the neurospheres were dissociated after reaching approximately 120 µm in diameter. To analyze the impact of the dissociation method on each NSC culture, in the next step of this study, two methods of dissociation were compared: enzymatic, known as the most commonly used, *vs.* the mechanical one. The viability was checked with two techniques: the calcein/Etdh-1 test as the live/dead assay and the LDH test as an intact/impaired indicator (Figure 9a and Figure 10a). First, we applied dissociation as the only factor in the standard culture medium (full medium) (Figure 9b and Figure 10b). Next, we correlated the method of dissociation with all culture medium composition variants (Figure 6a).

#### 3.3.1. Intra-Species Variability

In the live/dead assay, the cells cultured in a full medium demonstrated a significantly lower number (around 62%) of live rNSCs which were dissociated with the enzymatic method (Figure 9). However, no other significant differences were recorded either for rNSCs or for hNSCs and mNSCs.

In the LDH test, no significant changes in cells’ viability were observed in hNSCs when Day 1 and Day 7 of cultures were compared (Figure 9b).

The most remarkable differences were noted for mNSCs. On Day 1 of culture, LDH release was significantly more elevated in the cells dissociated enzymatically (13 ± 0.7% of total LDH release) than mechanically (9 ± 2% of total LDH release); however, the values changed dramatically after a week of culture, when the same cells’ viability seemed to decrease (7 ± 0.3% of total LDH release) and was much lower in enzymatically dissociated cells than in mechanically dissociated ones (30 ± 1% of total LDH release).

On rNSCs’ culture Day 1, we also observed a significantly elevated level of LDH release for enzymatically dissociated cells (7 ± 0.5%), however this effect was not visible after Day 7, and there were no remarkable differences between both presented dissociation methods.

When correlating different culture mediums with dissociation methods (Figure 6a), no significant changes were identified in hNSCs. However, in rodent cultures, the viability was found to significantly decrease after mechanical dissociation in the cells cultured in the glutamine-free medium (15 ± 4% and 30 ± 1% of total LDH release, respectively), and the effect was most pronounced on Day 7. Interestingly, 7 days after mechanical dissociation, mNSCs’ viability significantly decreased in the FGF medium (29 ± 0.6% of total LDH release), while the same observation was made for rNSCs in the EGF medium (42 ± 1% of total LDH release).

#### 3.3.2. Inter-Species Variability

There were no significant differences between the species in relation to the dissociation method and the day of culture recorded in the calcein/Ethd-1 test. However, dissociation method-dependent differences in LDH release during the culture were noted for all the analyzed species. The highest viability was noted for hNSCs regardless of the method and time of observation. The most significant differences were seen in enzymatic dissociation on Day 1 when the release of LDH was considerably more elevated in rodent NSCs than in hNSCs, and in rat more than in mouse cells. The relation was maintained after Day 7, however, the LDH release was less expressed. Conversely, a negative impact of mechanical dissociation was recorded most evidently in mNSCs.

### 3.4. hNSCs’ Characteristics

**Figure 11:**
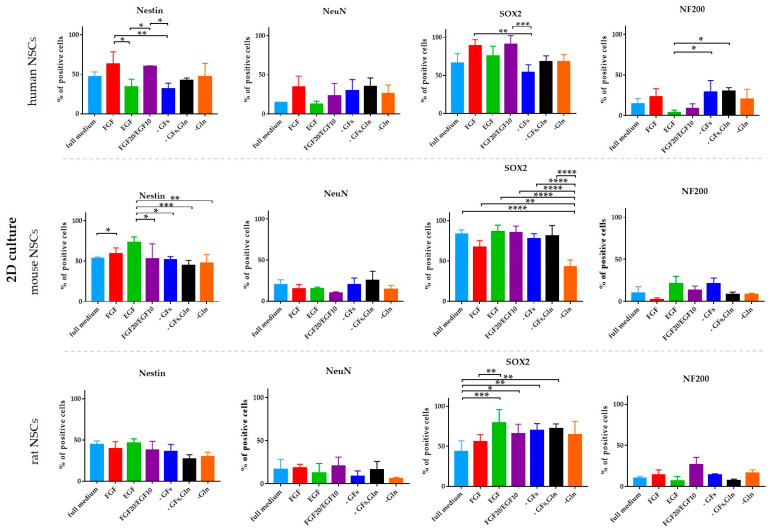
Neural differentiation of human, mouse, and rat NSCs cultured in 2D conditions. Quantitative analysis of neural (Nestin, SOX2) and neuronal (NeuN, NF200) markers. Cell culture conditions: 5% O_2_, 5% CO_2_, 37 °C. The results are presented as mean values of three experiments ± SEM. The differences were considered statistically significant when the *p*-value < 0.05. Statistical significance level: * for 0.01 < *p* < 0.05, ** for 0.001 < *p* < 0.01, *** for 0.0001 < *p* < 0.001, and **** for *p* < 0.0001.

**Figure 12:**
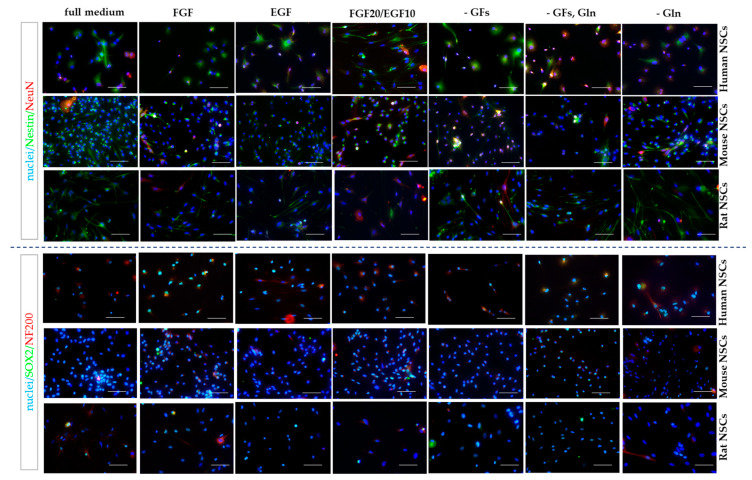
Neural differentiation of human, mouse, and rat NSCs in 2D conditions. Nestin, NeuN, SOX2_,_ and NF200 immunofluorescence analysis. Cell culture conditions: 5% O_2_, 5% CO_2_, 37 °C. Scale: 100 µm.

**Figure 13:**
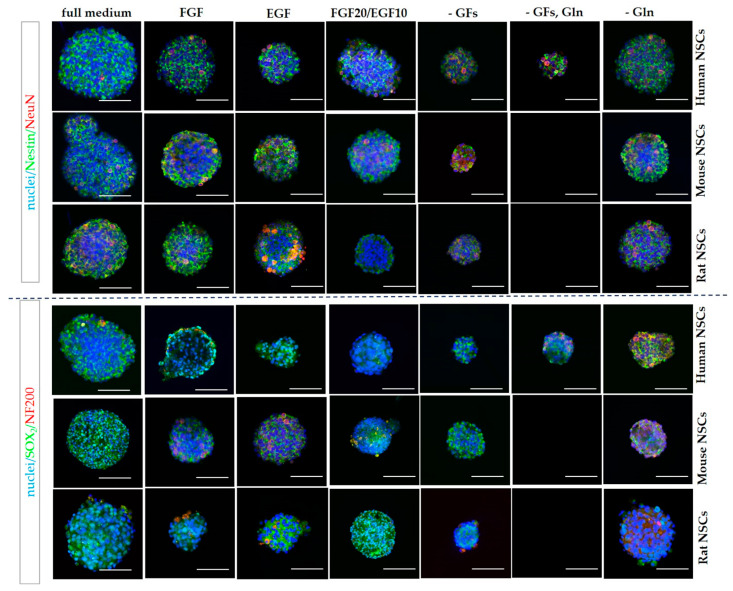
Neural differentiation of human, mouse, and rat NSCs in 3D conditions. Nestin, NeuN, SOX2_,_ and NF200 immunofluorescence analysis. Cell culture conditions: 5% O_2_, 5% CO_2_, 37 °C. Scale: 50 µm.

To analyze the neural differentiation stage of NSCs, the presence of selected neural (Nestin and SOX2) and neuronal (NeuN and NF200) markers was assessed using immunofluorescence staining. The quantitative analysis was performed in the 2D cell culture (Figure 11 and Figure 12). The IF-stained neurospheres treated with different culture media revealed their distinct impact on the presence of the aforementioned markers. Since a precise method of cell counting in the neurosphere is yet to be designed, we were only able to perform the qualitative analysis (Figure 13).

#### 3.4.1. Intra-Species Variability

In 2D culture of human NSCs, the most significant changes in the expression of Nestin and SOX2 markers were observed for all medium variants (Figure 11 and Figure 12). The highest expression of Nestin in NSCs was detected in hNSCs treated with FGF medium (62 ± 15%) and FGF10/EGF20 medium (60 ± 1%). The expression was significantly lower for the cells cultured in EGF (34 ± 10%) or without any growth factors (31 ± 7%). These results positively correlated with the expression of SOX2+ cells. The expression of neuronal markers was significantly lower in all tested variants, but the cells responded to the medium supplements in a similar pattern.

In the mNSCs’ 2D culture, a significant difference in Nestin presence was recorded between mNSCs cultured in FGF (59 ± 7%) and FGF10/EGF20 (52 ± 18%) medium, the medium without GFs (51 ± 4%), the GFs and glutamine variant (45 ± 6%), and glutamine-free medium (47 ± 10%). The highest expression of Nestin was observed in EGF medium (73 ± 7%). The most significant changes were seen for SOX2 expression, which was the lowest in the glutamine-free medium (42 ± 8%).

We did not observe any significant differences in Nestin, NeuN, and NF200 expression in the rNSC monolayer culture. The highest expression of SOX2 was noted in the EGF-treated cell medium (79 ± 16%). Interestingly, a significantly lower level of that marker was seen in rNSCs cultured in the full medium (43% ± 14%).

#### 3.4.2. Inter-Species Variability

**Figure 14:**
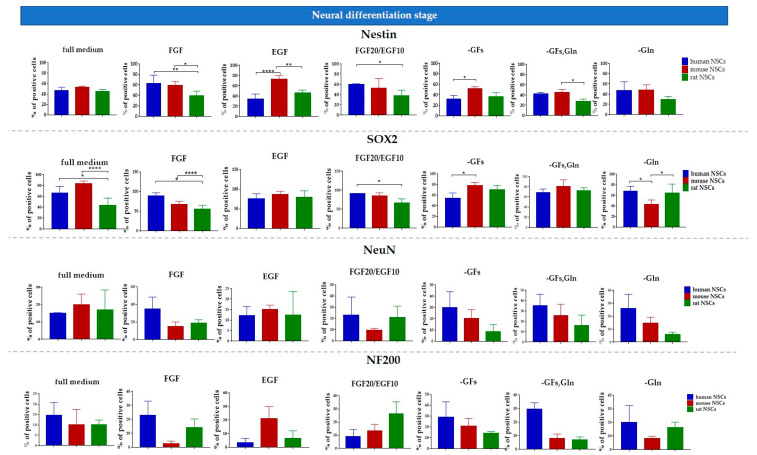
Neural differentiation of human NSCs in 2D culture. Quantitative analysis of neural (Nestin, SOX2) and neuronal (NeuN, NF200) markers. Cell culture conditions: 5% O_2_, 5% CO_2_, 37 °C; N = 3. The results are presented as mean values of three experiments ± SEM. The differences were considered statistically significant when the *p*-value < 0.05. Statistical significance level: * for 0.01 < *p* < 0.05, ** for 0.001 < *p* < 0.01 and **** for *p* < 0.0001.

The lowest expression of early neural markers was recorded in rNSCs. The oscillation in marker expression initiated by different supplements was mostly noted for SOX2 (Figure 14). This marker’s presence was significantly lower in rNSCs cultured in full medium (43 ± 14%) than in hNSCs and mNSCs (respectively, 66 ± 12% and 83 ± 5%). Similarly, the lowest percentage of SOX2+ cells was observed in rNSCs in FGF-treated medium, where the percentage of these cells oscillated up to 55 ± 9%. Conversely, in glutamine-free medium, a significantly lower expression of SOX2+ was seen in mNSCs (43 ± 9%) than in hNSCs (68 ± 9%) and rNSCs (65 ± 16%). Moreover, the SOX2+ level was significantly lower in rNSCs’ FGF20/EGF10 medium (65 ± 11%) than in hNSCs (91 ± 11%). The level of the medium without growth factors was more elevated in mNSCs (78 ± 6%) than in hNSCs (54 ± 10%). Moreover, clear differences in the presence of Nestin were identified between the species, with the most significant change being noted for the EGF medium variant. The highest percentage of positive cells was observed in mNSCs (73 ± 7%) and the value was significantly lower for both hNSCs (34 ± 10%) and rNSCs (46 ± 5%). A similar relation of Nestin’s presence was observed in FGF, FGF20/EGF10, and -GFs variants when compared with the changes in the SOX2 level between the species. We did not notice any significant changes between the species with regard to the presence of NeuN and NF200.

### 3.5. The Assessment of Migration of NSCs Grown as Neurospheres and Transferred to 2D Conditions

**Figure 15:**
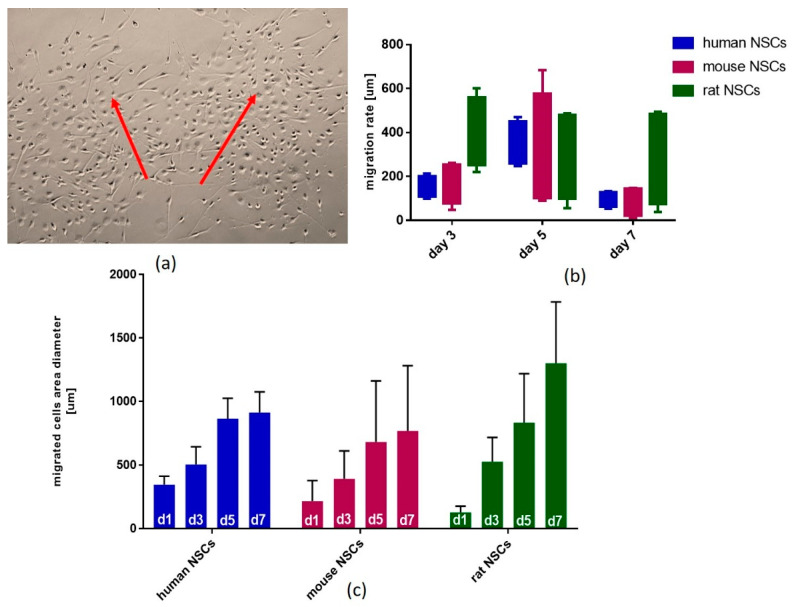
The migratory properties of the human, mouse, and rat NSCs. (**a**) Microscopic view of migrating human NSCs from 2 neurospheres (red arrows). (**b**) Migration rate of human, mouse, and rat NSCs on Days 3 (d3), 5 (d5), and 7 (d7) of culture. (**c**) Migrated cells’ area on Days 1, 3, 5, and 7 (d1, d3, d5, and d7, respectively). Cell culture conditions: 5% O_2,_ 5% CO_2_, 37 °C. N = 3.

To evaluate the functional properties of NSCs grown as neurospheres after changing the culture conditions into the monolayer, the neurospheres were transferred onto the coverslips coated with poly-L-lysine and laminin once they had reached the desired diameter (approximately 120–150 µm). After about 10–12 h, single cells began to migrate from the spheres. The migration of cells from the spheres was counted using the AxioVert A1 microscope, measuring the diameter of the field occupied by cells migrating from the spheres (Figure 15). Within the first 12 h of the start of the experiment, the cells were seen to adapt to the change in the spatial conditions, showing a high potential for migration. The migration rate changes during the culture were similar to those observed in human and mouse NSCs. The rate was the highest on Day 5 of culture, while in the rat NSCs the highest values were recorded on Day 3 (Figure 15b,c).

## 4. Discussion

Current treatments of some neurological diseases are still unsatisfactory. Even though the self-repair process is induced by the endogenously activated quiescent NSCs immediately after the brain injury, in most cases, it still proves to be insufficient. Therefore, a plethora of studies have searched how to enhance endogenous neuroprotection and neurorestoration or how to apply exogenous NSC treatment [16]. Some *in vivo* research on rats has shown that exogenous hNSCs do not replace the injured tissue but they actually recreate the microenvironment which can induce endogenous neurogenesis [22,23]. The benefits of cell therapies have already been revealed, however, over the years, scientists have been grappling with the issue of results’ interpretation as the reported outcomes seem to be laboratory-dependent and could have been affected at numerous levels, including a preclinical stage, for example, with regard to different species’ origin, or intra-specifically, due to different methods of isolation, banking, cryopreservation, expansion, long-term culture, or differentiation.

### 4.1. The Effect of Direct Cryopreservation on Human and Rodent NSCs’ Viability and Growth Potential

As mentioned above, the differences can be noticed in the variety of isolation protocols from the very beginning of the cell culture establishment. One of most important steps in further cell banking involves the use of an appropriate freezing/vitrifaction procedure. A well-optimized cryopreservation protocol is a prelude to a stable, intact supply of a reproducible NSC population. Even though the currently used methods of NSCs’ cryopreservation seem to be well-characterized, they still remain ambiguous. The protocols for cell freezing vary even intra-specifically, which can result in different cellular responses. Researchers are still striving to discover a better way to increase post-thawing cell survival. It has also been disputable whether shortening the isolation procedure by cell cryopreservation without previous culture cultivation could be as efficient as after standard protocol steps. Such an isolation method could be beneficial, especially in the establishment of primary cell cultures in which the isolation is performed fully/partially in hospital conditions. Nevertheless, our results confirmed the reports by Vescovi’s group [19,21], as we can also conclude that to perform successful derivation of NSC culture (regardless of cell origin), the cells should be cultured immediately after the isolation. Despite several thawing procedure verifications, the survival of hNSCs which were freshly cryopreserved after the isolation still seemed to be insufficient to continue the culture. The same effect was observed in rodent NSCs. The rNSCs and mNSCs that were freshly cryopreserved after isolation were characterized by a significantly lower cell viability than the cultivated ones. Thus, we recommend previous cell cultivation prior to their cryopreservation, even though it entails an immediate tissue transfer to the laboratory and the staff’s 24 h standby.

### 4.2. The Influence of the Medium Composition (Growth Factors, Glutamine) on the Viability, Proliferation, and Senescence of Human and Rodent NSCs

Nowadays, the establishment of physiological niche-like conditions seems to be the main goal in preclinical research as they are one of the major components is culture medium composition. An unquestionable significance of two main mitogens—bFGF and EGF—has been reported throughout the last 30 years, particularly in regard to NSC culture maintenance [24]. First and foremost, the mitogens have been proven to promote neurosphere formation [25]. However, some attention should be paid while interpreting their function individually or when comparing them with different species, as some studies have reported that they produce various effects depending on the type of culture (2D *vs*. 3D). Although neurosphere culture seems to be easier to perform, it has some limitations that can result in alterations of neurosphere frequency, which depends on many factors, e.g., medium composition, isolation method, dissociation process, or even the density of cultured cells [26,27,28,29]. We observed that the culture in EGF medium (without bFGF) decreased neurosphere formation, in mNSCs in particular, while in 2D culture this medium variant seemed to even strengthen their proliferation potential and maintained early neural markers’ presence (SOX2 and Nestin) in comparison to hNSCs and rNSCs. Reportedly, EGF induces cell division and increases the *Notch-1* intracellular domain level in neural progenitor cells, which was shown to be involved in the promotion of NSC survival and self-renewal in CNS development [24,30,31]. Moreover, embryonic mouse NSCs were shown to respond only to bFGF, while late embryonic and adult NSCs were found to be responsive to both bFGF and EGF [24,32,33,34]. Although our NSCs were not isolated in the early embryonic gestation stage, we observed slightly limited responsiveness to EGF in 3D culture. In addition, apart from the formed neurospheres, a monolayer of cells was observed in this variant and that could suggest a mix of EGF- and FGF-responsive cell populations in our culture. It is a combination of both bFGF and EGF that is most commonly used in culturing adult mouse subventricular zone cells, embryonic rat, and fetal human CNS cells; thus, it is difficult to compare such data with other groups [35]. On the other hand, limited formation of neurospheres was not seen in all our species in cell cultures whose medium lacked EGF. Thus, bFGF seemed to maintain this parameter for all presented species. It was previously shown that bFGF could promote the acquisition of EGF responsiveness in mNSCs [36]. However, the modulation of GFs concentration causes another difference to appear when compared to the most commonly used medium. We observed that the use of the FGF20/EGF10 variant of the medium produced a beneficial effect on cell proliferation, particularly in rodent NSCs. Such a concentration allowed NSCs of all origins to form neurospheres whose diameters were close to those of the full medium-treated NSCs. Moreover, this variant maintained one of the highest percentages of cells that were SOX2-positive in comparison to the full medium-treated groups, which was clearly visible in our rNSC culture. Moreover, another study reported that when EGF and bFGF were removed, despite the induced differentiation, stronger expression of cell survival-promoting genes—*igf-1* and *pdgfb*—was observed in mouse neural precursor cells, which could result in more effective engraftment of such cells with the host tissue [24]. Our 2D culture results showed a decreased number of neural marker Nestin+ cells after removing both GFs from all species, which was also previously reported in the available literature [24]. This could potentially be associated with their further differentiation; however, it was not subsequently investigated by us as it was not the subject matter of our study. We also confirmed the negative impact of both GFs removal on neurosphere formation as well as inhibition of proliferation potential for all species [25]. However, a much stronger reduction in forming neurospheres was seen for cultures cultivated in the -GFs, Gln medium. This variant was found to result in limited proliferation in all the species. Glutamine is known as an important source of energy [37], however, in many NSCs studies, researchers omit to add it to the medium or fail to mention it in the materials and methods section. This, in turn, could lead to various consequences regarding NSCs, which are yet to be clarified. It has been observed that withdrawal of glutamine in hair follicle stem cells could even maintain specialized stem cell niches via TORC-Akt signaling [38]. By regulating mTOR activity, translation, and autophagy, glutamine also coordinates the proliferation and growth of tumor stem cells, which could explain why the -GFs, Gln medium variant was observed here to be stronger than the medium without the GFs proliferation rate inhibition only [39]. Moreover, by affecting the aspartate-malate shuttle, glutamine can increase the NADPH/NADP(+) ratio and suppress oxidative stress as a result [40]. Therefore, it can also affect cell viability, which was detected particularly in our study on rodent cultures. The critical importance of glutamine metabolism for NSC maintenance was reported in the study on FoxO3 signaling [41]. The study demonstrated that the impaired glucose and glutamine metabolism compromised the proliferative potential of NPCs. Furthermore, regarding the NSCs’ differentiation stage, we observed that the -GFs, Gln medium variant led to a significantly higher percentage of Nestin+ mNSCs than rNSCs, but no differences in SOX2 were identified, which may suggest the impact of GFs and glutamine on further neural differentiation of mNSCs. We should also emphasize the drastic decrease which we observed in SOX2+ cells’ presence in mNSC culture in the medium deprived of glutamine only. Glutamine’s critical importance for the self-renewal and undifferentiated status maintenance of cells has already been demonstrated in mouse embryonic stem cells [42]. Thus, its loss could affect the expression of SOX2, which is known as a transcription factor that controls NSC long-term self-renewal and proliferation as well [43]. Our observation was followed by high-senescence formation and LDH release of mNSCs, which suggests the important role that glutamine played, especially in mouse-origin NSCs’ fate. Interestingly, glutamine significantly reduced the proliferation rate but did not remarkably affect the viability and senescence or the differentiation stage in our study’s hNSCs. Furthermore, the limitation of proliferation was stronger in this variant than in hNSCs cultured in a medium without both GFs and Glu, which indicates a possible interaction between glutamine activity and the presence of growth factors/supplements. However, this effect was not observed in our study’s rNSCs and mNSCs. This also suggests a different metabolic response in each species and should be further studied using more specialized tests. To sum up, the composition of the culture medium, and the presence of glutamine and growth factors in particular, is species-dependent, and it proves important for the cell proliferation, senescence, and viability.

### 4.3. The Influence of Dissociation Methods (Enzymatic and Mechanical) on the Viability, Proliferation, and Senescence of Human and Rodent NSCs

Another culture parameter indicated in the relevant literature includes the method of neurosphere dissociation. The enzymatic method has been established as a golden standard nowadays, as it provides better cell viability [44,45]. However, the most commonly used enzymes—accutase or trypsin—can impact cell viability to a different degree. In some studies, trypsin was demonstrated to induce cell membrane damage and cell death, while accutase seemed to increase cells’ survival, growth rate, and their viability [44,46,47]. In many studies, the enzymatic dissociation method was recommended to be applied in rodent and human NSC culture [44,48]. Nevertheless, benefits of the mechanical method of dissociation were also reported [49,50]. Even though this procedure seems to be quite aggressive, it was reported to provide a higher expansion rate in hNSCs when compared to the enzymatic method [51]. As the studies on the impact of mechanical dissociation on NSC fate are scarce, we decided to analyze basic parameters—the number of live cells and the LDH release. The assessment of the LDH release after 7 days of culture confirmed better cell viability in the enzymatically dissociated cells, especially in mNSCs. These data, however, differed from our results obtained after Day 1 of culture in mNSCs as well as in rNSCs, where the LDH release was significantly elevated after enzymatic dissociation, which suggests that although directly after 24 h of this procedure, the enzymatic method could affect the cells more drastically, the cell recovery after 7 days of culture is significantly higher than after the mechanical procedure. This also shows the importance of the day of the analysis performance. Moreover, the results appeared to be species-dependent. We observed the highest LDH release in rNSCs after enzymatic dissociation, however, the highest release was noted for mNSCs after the mechanical method. After the analysis was performed, yet another question arose: is it reliable to analyze the parameters individually? For example, no considerable differences in the number of calcein+ cells between the species were observed in response to different dissociation methods, which could suggest that the viability of cells is the same for both presented methods. However, we did observe statistically significant changes in LDH release, which may suggest more substantial cell membrane damage in some of the culture variants. We believe that the obtained results should be followed by further, more specified proliferation/senescence analyses, which will be our further issue of interest. Nevertheless, the dissociation method selection already seems to be species-dependent and to have a key impact on the parameters achieved in long-term culture.

### 4.4. The Influence of Spatial Conditions (3D and 2D Culture) on the Viability, Proliferation, and Senescence of Human and Rodent NSCs

NSCs can be cultured either as a monolayer (2D) or in a suspension (3D). Both methods have already shown several advantages and disadvantages, which should be analyzed with regard to the future NSCs’ application. The 2D culture has been shown to provide conditions that allow for a more homogeneous population of NSCs, which also proliferate faster [19,20]. However, it does not adequately recreate the microenvironment of physiological 3D niche and cell-to-cell interactions; thus, it can lead to misleading results of *in vivo* responses [24]. Moreover, the growth potential and self-renewal of NCSs seem to be higher in 3D culture [52]. Thus, transplantation of NSCs as neurospheres to a damaged CNS seems to produce multiple benefits.

## 5. Conclusions

To summarize, we outlined the critical importance of the NSCs’ culture conditions’ optimization, and the following points seem to be of particular significance:

- Direct cultivation of NSCs before cryopreservation.
- Proper concentration of growth factors (bFGF and EGF) in the medium, which we estimated at 20 ng/mL for both bFGF and EGF.
- Presence of glutamine in the medium.
- Enzymatic method of neurosphere dissociation.

This would also simplify the comparison of the obtained results with a larger number of the available studies. We also showed that the migratory potential of each species changes over time, which could be useful while interpreting experiments *in vivo*.

It should be emphasized that possible reasons for the intra-species differences in proliferation, senescence, LDH activity, or the differentiation stage could be associated with the method, region, and time of NSC isolation. So far, NSCs have been derived from the dental gyrus of the hippocampi, the SVZ region, the olfactory bulb, the subcallosal zone underlying the corpus callosum, as well as the spinal cord of the embryonic, newborn, and adult rodent CNS. They have also been derived from developing and adult human brains [20,53,54]. Their further expansion *in vitro* is possible thanks to the use of mitogens [24,55] and propagating genes [56,57]. The NSCs isolated from embryonic, fetal, and adult brains are known to show different characteristics; moreover, they differ between rodent and human origin. However, the comparison of the three species analyzed here has only been scarcely discussed in the available literature so far. What appears to be a crucial step after NSC isolation is the verification of their stemness properties, including proliferation ability, self-renewal capacity, functional stability, and multipotentiality [53,58].

**Figure 16:**
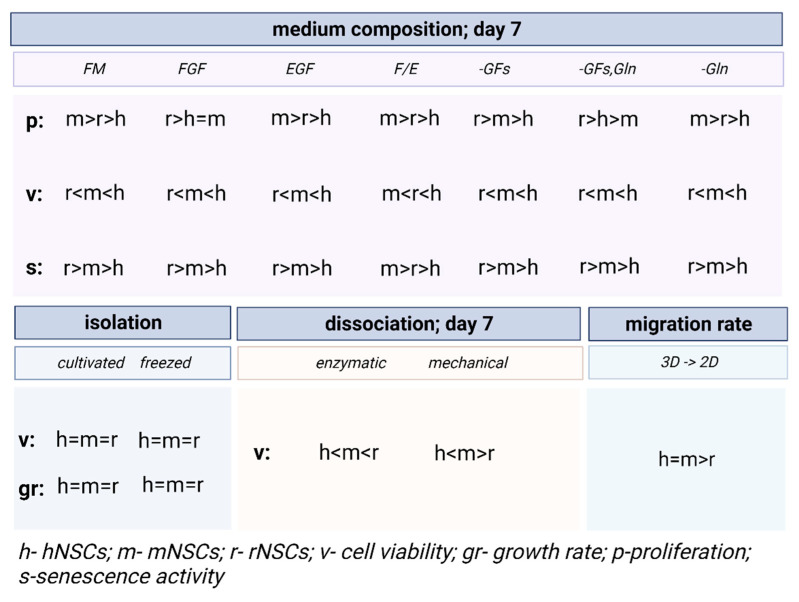
The summary of selected inter-species variability in different culture conditions.

**Figure 17:**
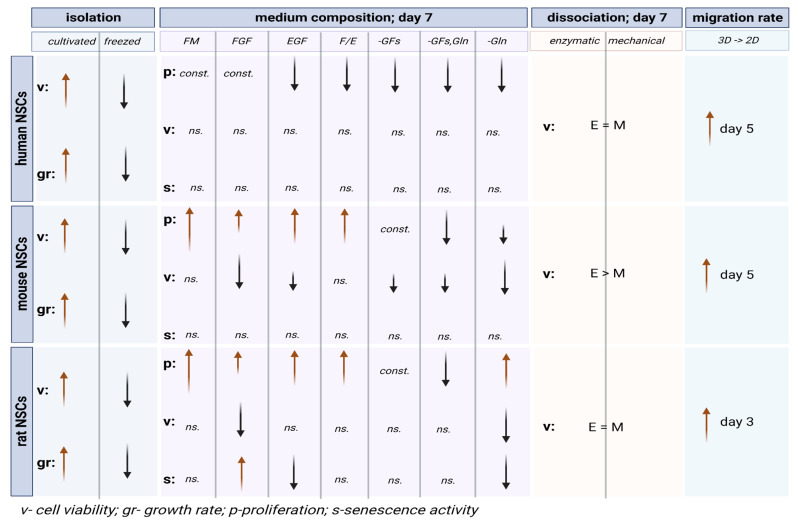
The summary of selected intra-species variability in different culture conditions.

Moreover, with no available data on the medium composition’s impact on human NSCs, we should not be directly inspired by the experimental results obtained on rodent NSCs as there is a multitude of inter-species differences. Although the hNSC proliferation rate was lower in comparison to rodent NSCs, they displayed better cell viability and decreased senescence (Figure 16). Moreover, hNSCs were shown to require a complete medium to maintain their proliferative abilities, while rodent NSCs appeared less demanding and responded to the lack of both growth factors and glutamine to decrease proliferation (Figure 17). According to our results obtained in the most commonly used medium, regarding neurosphere formation potential and the neural differentiation stage, more similarities were observed between hNSCs and rNSCs than between hNSCs and mNSCs; however, hNSCs were more similar to mNSCs with regard to senescence, cell viability, and migratory potential. Another issue regarding further studies using an *in vivo* model is that we should keep in mind the plethora of developmental and functional differences between present neurotraumatic and neurodegenerative animal models. Various expansions of the neocortex, neuronal subtypes, and human-specific aspects of gene regulation and expression affect the abilities of these models to recapitulate the human brain and the pathological mechanisms of neurological disorders, especially those influencing intellectual diseases [59].

## Figures and Tables

**Table 1:** The medium composition, coating, and seeding density used in the following experiments.

	**2D Culture**	**3D Culture**
Medium composition	DMEM/F12 (Gibco, Thermo Fisher Scientific, Waltham, MA, USA) GlutaMAX^®^ (1%, Gibco) Penicillin/streptomycin (1%, Gibco) Heparin (0.1%, Sigma-Aldrich, Saint Louis, MO, USA) N2 supplement ^®^ (1%, Gibco) B27 supplement^®^ (2%, Gibco) EGF (20 ng/mL, Gibco) bFGF (20 ng/mL, Gibco)	
Coating	poli-L-lysine + laminin	The non-adhesive coating on NunclonSphera^®^ plates and t75 flasks (Nunc, Thermo Scientific™)
Seeding density	40,000 cells/cm^2^	1 × 10^4^ cells/cm^2^

**Table 2:** Primary antibodies used for the experiments.

Antibody	Catalog Number	Source	Isotype	Dilution	Manufacturer
**anti-Nestin**	SAB4200347	Rabbit polyclonal	IgG	1:500	Sigma-Aldrich
**anti-NeuN**	MAB377	Mouse monoclonal	IgM	1:50	Millipore
**anti-NF200**	N0142	Mouse monoclonal	IgG1	1:800	Sigma-Aldrich
**anti-SOX2**	SAB5700644	Rabbit polyclonal	IgG	1:150	Sigma-Aldrich

**Table 3:** Secondary antibodies used for the experiments.

Antibody	Fluorochrome	Catalog Number	Isotype	Dilution	Manufacturer
**Alexa Fluor Goat (anti-rabbit)**	Alexa 488	A11034	IgG	1:1000	Life Technologies
**Alexa FluorGoat (anti-mouse)**	Alexa 546	A21123	IgG1	1:1000	Life Technologies
**Alexa FluorGoat (anti-mouse)**	Alexa 546	A21045	IgM	1:1000	Life Technologies

## Data Availability

Not applicable.

## Abbreviations

NSCs	neural stem cells
hNSCs	human neural stem cells
rNSCs	rat neural stem cells
mNSCs	mouse neural stem cells
LDH	lactate dehydrogenase
HBSS	Hanks’ Balanced Salt Solution
DMSO	dimethyl sulfoxide
CNS	central nervous system
bFGF	basic fibroblast growth factor
EGF	epidermal growth factor
NGF	nerve growth factor
BDNF	brain-derived neurotrophic factor
VEGF	vascular endothelial growth factor
IGF	insulin-like growth factor

## References

[1] Lippert T., Gelineau L., Napoli E., Borlongan C.V. (2018). Harnessing neural stem cells for treating psychiatric symptoms associated with fetal alcohol spectrum disorder and epilepsy. Prog. Neuro-Psychopharmacol. Biol. Psychiatry.

[2] Beattie R., Hippenmeyer S. (2017). Mechanisms of radial glia progenitor cell lineage progression. FEBS Lett..

[3] Rakic P. (2009). Evolution of the neocortex: A perspective from developmental biology. Nat. Rev. Neurosci..

[4] Yan Y., Shin S., Jha B.S., Liu Q., Sheng J., Li F., Zhan M., Davis J., Bharti K., Zeng X. (2013). Efficient and Rapid Derivation of Primitive Neural Stem Cells and Generation of Brain Subtype Neurons from Human Pluripotent Stem Cells. STEM CELLS Transl. Med..

[5] Ferrari D., Gelati M., Profico D.C., Vescovi A.L. (2018). Human Fetal Neural Stem Cells for Neurodegenerative Disease Treatment. Results Probl. Cell Differ..

[6] Pluchino S., Zanotti L., Deleidi M., Martino G. (2005). Neural stem cells and their use as therapeutic tool in neurological disorders. Brain Res..

[7] Zhou T., Yuan Z., Weng J., Pei D., Du X., He C., Lai P. (2021). Challenges and advances in clinical applications of mesenchymal stromal cells. J. Hematol. Oncol..

[8] Mitrecic D., Nicaise C., Klimaschewski L., Gajovic S., Bohl D., Pochet R. (2012). Genetically modified stem cells for the treatment of neurological diseases. Front. Biosci..

[9] Mitrečić D. (2011). Current Advances in Intravascular Administration of Stem Cells for Neurological Diseases: A New Dose of Rejuvenation Injected. Rejuvenation Res..

[10] Hribljan V., Salamon I., Đemaili A., Alić I., Mitrečić D. (2018). Transplantation of neural stem cells in the mouse model of ischemic brain stroke and expression of genes involved in programmed cell death. Croat. Med. J..

[11] Martino G., Pluchino S. (2006). The therapeutic potential of neural stem cells. Nat. Rev. Neurosci..

[12] Willis C.M., Nicaise A.M., Peruzzotti-Jametti L., Pluchino S. (2020). The Neural Stem Cell Secretome and Its Role in Brain Repair.

[13] Kaminska A., Radoszkiewicz K., Rybkowska P., Wedzinska A., Sarnowska A. (2022). Interaction of Neural Stem Cells (NSCs) and Mesenchymal Stem Cells (MSCs) as a Promising Approach in Brain Study and Nerve Regeneration. Cells.

[14] Marei H.E., Casalbore P., Althani A., Coccè V., Cenciarelli C., Alessandri G., Brini A.T., Parati E., Bondiolotti G., Pessina A. (2019). Human Olfactory Bulb Neural Stem Cells (Hu-OBNSCs) Can Be Loaded with Paclitaxel and Used to Inhibit Glioblastoma Cell Growth. Pharmaceutics.

[15] Rezk S., Lashen S., El-Adl M., Elshopakey G.E., Elghareeb M.M., Hendam B.M., Caceci T., Cenciarelli C., Marei H.E. (2022). Effects of Rosemary Oil (Rosmarinus officinalis) supplementation on the fate of the transplanted human olfactory bulb neural stem cells against ibotenic acid-induced neurotoxicity (Alzheimer model) in rat. Metab. Brain Dis..

[16] Huang L., Zhang L. (2018). Neural stem cell therapies and hypoxic-ischemic brain injury. Prog. Neurobiol..

[17] Tuazon J.P., Castelli V., Lee J.-Y., Desideri G.B., Stuppia L., Cimini A.M., Borlongan C.V. (2019). Neural Stem Cells. Adv. Exp. Med. Biol..

[18] Gao Q., Liao L.-Y., Lau B.W.-M., Sánchez-Vidaña D.I. (2019). Exogenous neural stem cell transplantation for cerebral ischemia. Neural Regen. Res..

[19] Ferrari D., Binda E., De Filippis L., Vescovi A.L. (2010). Isolation of Neural Stem Cells from Neural Tissues Using the Neurosphere Technique. Curr. Protoc. Stem Cell Biol..

[20] Vescovi A.L., Parati E.A., Gritti A., Poulinb P., Ferrariob M., Wankec E., Schoellera P.F., Cova L., Panliliob M.A., Colombod A. (1999). Isolation and Cloning of Multipotential Stem Cells from the Embryonic Human CNS and Establishment of Transplantable Human Neural Stem Cell Lines by Epigenetic Stimulation. Exp. Neurol..

[21] Gelati M., Profico D., Projetti-Pensi M., Muzi G., Sgaravizzi G., Vescovi A.L. (2013). Culturing and Expansion of “Clinical Grade” Precursors Cells from the Fetal Human Central Nervous System. Methods Mol. Biol..

[22] Park D.-H., Eve D.J., Sanberg P.R., Musso J., Bachstetter A.D., Wolfson A., Schlunk A., Baradez M.-O., Sinden J.D., Gemma C. (2010). Increased Neuronal Proliferation in the Dentate Gyrus of Aged Rats Following Neural Stem Cell Implantation. Stem Cells Dev..

[23] Kim J.-H., Lee J.-E., Kim S.U., Cho K.-G. (2010). Stereological Analysis on Migration of Human Neural Stem Cells in the Brain of Rats Bearing Glioma. Neurosurgery.

[24] Reynolds B.A., Weiss S. (1992). Generation of Neurons and Astrocytes from Isolated Cells of the Adult Mammalian Central Nervous System. Science.

[25] Deleyrolle L.P., Reynolds B.A. (2009). Isolation, Expansion, and Differentiation of Adult Mammalian Neural Stem and Progenitor Cells Using the Neurosphere Assay. Methods Mol. Biol..

[26] Soares R., Ribeiro F.F., Lourenço D.M., Rodrigues R.S., Moreira J.B., Sebastião A.M., Morais V.A., Xapelli S. (2020). Isolation and Expansion of Neurospheres from Postnatal (P1−3) Mouse Neurogenic Niches. JoVE J. Vis. Exp..

[27] Azari H., Sharififar S., Rahman M., Ansari S., Reynolds B.A. (2011). Establishing Embryonic Mouse Neural Stem Cell Culture Using the Neurosphere Assay. J. Vis. Exp..

[28] Azari H., Louis S.A., Sharififar S., Vedam-Mai V., Reynolds B.A. (2011). Neural-Colony Forming Cell Assay: An Assay To Discriminate Bona Fide Neural Stem Cells from Neural Progenitor Cells. J. Vis. Exp..

[29] Reynolds B.A., Rietze R.L. (2005). Neural stem cells and neurospheres—re-evaluating the relationship. Nat. Methods.

[30] Campos L.S., Decker L., Taylor V., Skarnes W. (2006). Notch, Epidermal Growth Factor Receptor, and β1-Integrin Pathways Are Coordinated in Neural Stem Cells. J. Biol. Chem..

[31] Lathia J.D., Mattson M.P., Cheng A. (2008). Notch: From neural development to neurological disorders. J. Neurochem..

[32] Kilpatrick T., Bartlett P. (1995). Cloned multipotential precursors from the mouse cerebrum require FGF-2, whereas glial restricted precursors are stimulated with either FGF-2 or EGF. J. Neurosci..

[33] Tropepe V., Sibilia M., Cirunacd B.G., Rossantced J., Wagner E.F., Van Der Kooy D. (1999). Distinct Neural Stem Cells Proliferate in Response to EGF and FGF in the Developing Mouse Telencephalon. Dev. Biol..

[34] Arsenijevic Y., Weiss S., Schneider B., Aebischer P. (2001). Insulin-Like Growth Factor-I Is Necessary for Neural Stem Cell Proliferation and Demonstrates Distinct Actions of Epidermal Growth Factor and Fibroblast Growth Factor-2. J. Neurosci..

[35] Louis S.A., Mak C.K.H., Reynolds B.A. (2013). Methods to Culture, Differentiate, and Characterize Neural Stem Cells from the Adult and Embryonic Mouse Central Nervous System. Methods Mol. Biol..

[36] Ciccolini F., Svendsen C.N. (1998). Fibroblast Growth Factor 2 (FGF-2) Promotes Acquisition of Epidermal Growth Factor (EGF) Responsiveness in Mouse Striatal Precursor Cells: Identification of Neural Precursors Responding to both EGF and FGF-2. J. Neurosci..

[37] DeBerardinis R.J., Mancuso A., Daikhin E., Nissim I., Yudkoff M., Wehrli S., Thompson C.B. (2007). Beyond aerobic glycolysis: Transformed cells can engage in glutamine metabolism that exceeds the requirement for protein and nucleotide synthesis. Proc. Natl. Acad. Sci. USA.

[38] Kim C.S., Ding X., Allmeroth K., Denzel M.S., Eming S.A., Wickströ S.A. (2020). Glutamine Metabolism Controls Stem Cell Fate Reversibility and Long-Term Maintenance in the Hair Follicle. Cell Metab..

[39] Nicklin P., Bergman P., Zhang B., Triantafellow E., Wang H., Nyfeler B., Yang H., Hild M., Kung C., Wilson C. (2009). Bidirectional Transport of Amino Acids Regulates mTOR and Autophagy. Cell.

[40] Son J., Lyssiotis C.A., Ying H., Wang X., Hua S., Ligorio M., Perera R.M., Ferrone C.R., Mullarky E., Shyh-Chang N. (2013). Glutamine supports pancreatic cancer growth through a KRAS-regulated metabolic pathway. Nature.

[41] Yeo H., Lyssiotis C., Zhang Y., Ying H., Asara J.M., Cantley L.C., Paik J.-H. (2013). FoxO3 coordinates metabolic pathways to maintain redox balance in neural stem cells. EMBO J..

[42] Ryu J.M., Lee S.H., Seong J.K., Han H.J. (2015). Glutamine contributes to maintenance of mouse embryonic stem cell self-renewal through PKC-dependent downregulation of HDAC1 and DNMT1/3a. Cell Cycle.

[43] Pagin M., Pernebrink M., Giubbolini S., Barone C., Sambruni G., Zhu Y., Chiara M., Ottolenghi S., Pavesi G., Wei C.-L. (2021). Sox2 Controls Neural Stem Cell Self-Renewal Through a Fos-Centered Gene Regulatory Network. Stem. Cells.

[44] Wachs F.-P., Couillard-Despres S., Engelhardt M., Wilhelm D., Ploetz S., Vroemen M., Kaesbauer J., Uyanik G., Klucken J., Karl C. (2003). High Efficacy of Clonal Growth and Expansion of Adult Neural Stem Cells. Lab. Investig..

[45] Gil-Perotín S., Duran-Moreno M., Cebrián-Silla A., Ramírez M., García-Belda P., García-Verdugo J.M. (2013). Adult Neural Stem Cells from the Subventricular Zone: A Review of the Neurosphere Assay. Anat. Rec..

[46] Zhou H., Yang H., Liu L., Li X., Pan B., Fu Z., Chu T., Liu J., Kang Y., Ning G. (2020). A modified protocol for the isolation, culture, and cryopreservation of rat embryonic neural stem cells. Exp. Ther. Med..

[47] Li T., Li C., Zhang C.-Y., Zhao J. (2015). Effect of accutase or trypsin dissociation on the apoptosis of human striatum-derived neural stem cells. Zhongguo Yi Xue Ke Xue Yuan Xue Bao.

[48] Zheng K., Liu T.-Q., Dai M.-S., Ge D., Li X.-Q., Ma X.-H., Cui Z.-F. (2006). Comparison of different culture modes for long-term expansion of neural stem cells. Cytotechnology.

[49] Sen A., Kallos M.S., Behie L.A. (2004). New Tissue Dissociation Protocol for Scaled-up Production of Neural Stem Cells in Suspension Bioreactors. Tissue Eng..

[50] Azari H., Rahman M., Sharififar S., Reynolds B.A. (2010). Isolation and Expansion of the Adult Mouse Neural Stem Cells Using the Neurosphere Assay. JoVE J. Vis. Exp..

[51] Martín-Ibáñez R., Guardia I., Pardo M., Herranz C., Zietlow R., Vinh N.-N., Rosser A., Canals J.M. (2017). Insights in spatio-temporal characterization of human fetal neural stem cells. Exp. Neurol..

[52] Sun T., Wang X., Xie S., Zhang D., Wang X., Li B., Ma W., Xin H. (2011). A comparison of proliferative capacity and passaging potential between neural stem and progenitor cells in adherent and neurosphere cultures. Int. J. Dev. Neurosci..

[53] Gritti A., Frölichsthal-Schoeller P., Galli R., Parati E.A., Cova L., Pagano S.F., Bjornson C.R., Vescovi A.L. (1999). Epidermal and Fibroblast Growth Factors Behave as Mitogenic Regulators for a Single Multipotent Stem Cell-Like Population from the Subventricular Region of the Adult Mouse Forebrain. J. Neurosci..

[54] Sanai N., Tramontin A.D., Quiñones-Hinojosa A., Barbaro N.M., Gupta N., Kunwar S., Lawton M.T., McDermott M.W., Parsa A.T., García-Verdugo J.M. (2004). Unique astrocyte ribbon in adult human brain contains neural stem cells but lacks chain migration. Nature.

[55] Gritti A., Parati E., Cova L., Frolichsthal P., Galli R., Wanke E., Faravelli L., Morassutti D., Roisen F., Nickel D. (1996). Multipotential stem cells from the adult mouse brain proliferate and self-renew in response to basic fibroblast growth factor. J. Neurosci..

[56] Ryder E.F., Snyder E.Y., Cepko C.L. (1990). Establishment and characterization of multipotent neural cell lines using retrovirus vector-mediated oncogene transfer. J. Neurobiol..

[57] De Filippis L., Lamorte G., Snyder E.Y., Malgaroli A., Vescovi A.L. (2007). A Novel, Immortal, and Multipotent Human Neural Stem Cell Line Generating Functional Neurons and Oligodendrocytes. STEM CELLS.

[58] Grochowski C., Radzikowska E., Maciejewski R. (2018). Neural stem cell therapy—Brief review. Clin. Neurol. Neurosurg..

[59] Zhao X., Bhattacharyya A. (2018). Human Models Are Needed for Studying Human Neurodevelopmental Disorders. Am. J. Hum. Genet..

